# Case Report: Compound Heterozygous Phosphatidylinositol-Glycan Biosynthesis Class N (*PIGN*) Mutations in a Chinese Fetus With Hypotonia-Seizures Syndrome 1

**DOI:** 10.3389/fgene.2020.594078

**Published:** 2020-10-27

**Authors:** Shi-qi Xiao, Mei-hui Li, Yi-lin Meng, Chuang Li, Hai-long Huang, Cai-xia Liu, Yuan Lyu, Quan Na

**Affiliations:** ^1^Department of Nursing, Shengjing Hospital of China Medical University, Shenyang, China; ^2^Department of Obstetrics and Gynecology, Shengjing Hospital of China Medical University, Shenyang, China; ^3^Key Laboratory of Maternal-Fetal Medicine of Liaoning Province, Key Laboratory of Obstetrics and Gynecology of Higher Education of Liaoning Province, Shenyang, China

**Keywords:** *PIGN*, multiple congenital anomalies-hypotonia-seizures syndrome 1, reproduction guidance, prenatal diagnosis, glycosylphosphatidylinositol-anchor biosynthesis pathway

## Abstract

Multiple congenital anomalies-hypotonia-seizures syndrome 1 (MCAHS1) caused by phosphatidylinositol-glycan biosynthesis class N (*PIGN)* mutations is an autosomal recessive disease involving many systems of the body, such as the urogenital, cardiovascular, gastrointestinal, and central nervous systems. Here, compound heterozygous variants NM_012327.6:c.2427-2A > G and c.963G > A in *PIGN* were identified in a Chinese proband with MCAHS1. The features of the MCAHS1 family proband were evaluated to understand the mechanism of the *PIGN* mutation leading to the occurrence of MCAHS1. Ultrasound was conducted to examine the fetus, and his clinical manifestations were evaluated. Genetic testing was performed by whole-exome sequencing and the results were verified by Sanger sequencing of the proband and his parents. Reverse transcription-polymerase chain reaction was performed, and the products were subjected to Sanger sequencing. Quantitative PCR (Q-PCR) was conducted to compare gene expression between the patient and wild-type subjects. The compound heterozygous mutation NM_012327.6:c.2427-2A > G and c.963G > A was identified by whole-exome sequencing and was confirmed by Sanger sequencing. The NM_012327.6:c.2427-2A > G mutation led to skipping of exon 26, which resulted in a low expression level of the gene, as measured by Q-PCR. These findings provided a basis for genetic counseling and reproduction guidance in this family. Phenotype-genotype correlations may be defined by an expanded array of mutations.

## Introduction

Multiple congenital anomalies-hypotonia-seizures syndrome 1 (MCAHS1) is an autosomal recessive disease characterized by hypotonia, seizures, facial anomalies, and developmental delay, and involves various systems, such as the gastrointestinal tract, cardiovascular system, urogenital system, and central nervous system (Khayat et al., 2016). The etiology of MCAHS1 is based on mutations in phosphatidylinositol-glycan biosynthesis class N (*PIGN*). *PIGN* is one of more than 20 genes involved in the glycosylphosphatidylinositol (GPI)-anchor biosynthesis pathway (Couser et al., 2015). To date, eight genes [phosphatidylinositol glycan class A (*PIGA*), phosphatidylinositol glycan anchor biosynthesis class L (*PIGL*), phosphatidylinositol glycan anchor biosynthesis class M (*PIGM*), *PIGN*, phosphatidylinositol glycan anchor biosynthesis class O (*PIGO*), phosphatidylinositol glycan anchor biosynthesis class V (*PIGV*), phosphatidylinositol glycan anchor biosynthesis class T (*PIGT*), and post-GPI attachment to proteins 2 (*PGAP2*)] in this pathway are reported to be associated with neural abnormalities (Khayat et al., 2016). As *PIGN* is expressed in many tissues, several body systems are affected by mutations in this gene, leading to severe developmental delay (Couser et al., 2015). Here, we report a fetus with head and neck hygroma that subsequently disappeared and tetralogy of Fallot in a Chinese family. To clarify the genotype-phenotype relationship and provide a basis for genetic counseling, whole-exome sequencing (WES), Sanger sequencing, and reverse transcription-polymerase chain reaction (RT-PCR) were performed. The compound heterozygous *PIGN* mutation NM_012327.6:c.2427-2A > G was identified in the fetus. The transcription product of c.2427-2A > G mutation was found to result in skipping of exon 26.

## Case Description

### Pedigree and Clinical Evaluations

Gestational ultrasound at 12 weeks and 3 days revealed a neck hygroma approximately 32 mm × 17 mm in size, trunk edema, as well as nuchal translucency of 6.6 mm. Other measurements included biparietal diameter that was approximately 21 mm, femur length that was approximately 7 mm, and crown-lump length that was approximately 70 mm. The head and neck hygroma disappeared at 16 weeks. At 24 weeks, systematic ultrasound revealed tetralogy of Fallot in the fetus. Amniocentesis revealed a normal karyotype. After explaining the disease condition and related risks to the patient’s parents, they voluntarily requested the induction of labor. This study was approved by the ethics committee of Shengjing Hospital of China Medical University (ethics approval number: 2013PS33K).

### Identification and Functional Characterization of Variants

Peripheral blood samples were collected from the proband’s family members and tissue was collected from the proband. Genomic DNA was extracted using a TIANamp Blood DNA Kit (TIANGEN, Beijing, China) and subjected to WES. Targeted exon sequences plus flanking sequences were captured and enriched using an array-based hybridization chip (xGen Exome Research Panel v1.0, Integrated DNA Technologies, Coralville, IA, United States) followed by HiSeq X10 sequencing (Illumina, San Diego, CA, United States). All variants on autosomes and sex chromosomes were annotated using the Annotate Variation tool. The pathogenicity of variants was annotated using the Human Gene Mutation Database^1^, ClinVar database^2^, and Standards and Guidelines for the Interpretation of Sequence Variants of the American College of Medical Genetics and Genomics (ACMG) (Richards et al., 2015). A series of *in silico* impact score procedures, including Mendelian Clinically Applicable Pathogenicity^3^, Sorting Intolerant from Tolerant^4^, Polymorphism Phenotyping v2^5^, Likelihood Ratio Test^6^, MutationTaster^7^, Combined Annotation Dependent Depletion (CADD)^8^, Functional Analysis through Hidden Markov Models^9^, and Protein Variation Effect Analyzer^10^ were used to prioritize all variants according to the ACMG guidelines. Variants with minor allele frequencies <0.01 in any of the databases used [Single Nucleotide Polymorphism Database, Exome Aggregation Consortium (ExAC), 1000 Genomes Project, Genome Aggregation Database (gnomAD), and an in-house database] were selected. The WES results were validated by Sanger sequencing of the patient and his family members. RT-PCR using total RNA extracted from fetal tissue was performed. Briefly, total RNA was extracted using the RNeasy Plus Mini kit (QIAGEN, Hilden, Germany) from lymphoblastoid cell lines with or without incubation in 30 μM cycloheximide (Sigma, St. Louis, MO, United States) for 4 h. Four micrograms of total RNA were subjected to reverse transcription, and 2 μL cDNA was used for PCR. Primer sequences were ex29-F (5′-TCAAGCCAGCTGCCATAATC-3′) and ex22-R (5′-GTGCCACTACTGAGTTCTCCA-3′). PCR products were electrophoresed on a 10% polyacrylamide gel, bands were purified using an E.Z.N.A. poly-Gel DNA Extraction kit (Omega Bio-Tek, Norcross, GA, United States), and sequenced. RT-PCR products were sequenced using Sanger sequencing and quantitative PCR (Q-PCR) of the gene transcripts was performed.

**FIGURE 1:**
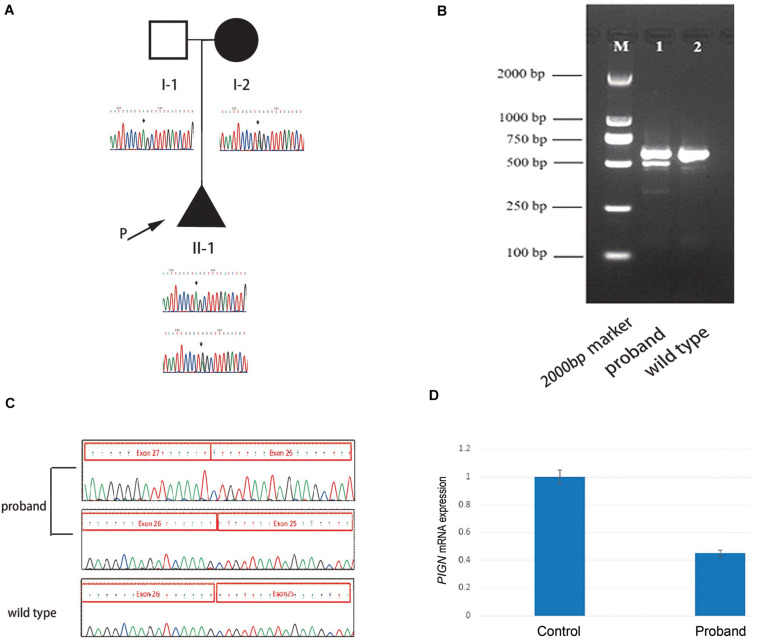
**(A)** Pedigree of the family and Sanger sequencing results of each family member. **(B)** The proband’s polymerase chain reaction (PCR) product contains two electrophoretic bands: 569 and 493 bp; the wild-type has only one band: 596 bp. **(C)** Sanger sequencing results of the reverse transcription (RT)-PCR product of the fetus and the wild-type, respectively. **(D)** Quantitative-PCR of phosphatidylinositol-glycan biosynthesis class N (*PIGN*) reveals lower expression in the patient than in the normal control.

High throughput sequencing of the patient revealed a synonymous mutation: c.963G > A/p.Gln321 = showing compound heterozygosity with a novel splicing mutation: c.2427-2 A > G. Sanger sequencing of the patient and his family members validated these results (Figure 1A). Mutations in *PIGN* were acquired from both parents separately. According to the ACMG standards, both mutations were defined as likely pathogenic. c.963G > A/p.Gln321 = is a synonymous mutation located in exon 10. The two mutations had a very low carrying rate in the ExAC and gnomAD databases (Table 1). c.2427-2 A > G is a splicing mutation located in intron 25 and may lead to abnormal mRNA splicing that influences protein expression. This mutation is predicted to cause loss of function of the protein. Sanger sequencing of the RT-PCR product revealed that the splicing mutation c.2427-2 A > G led to skipping of exon 26 (Figures 1B,C). A hazardous assessment of the mutation: c.963G > A/p.Gln321 = was conducted. The CADD score was 32, Genomic Evolutionary Rate Profiling (GERP) score was 5.56, DANN score was 0.995, and the PhyloP100way vertebrate Score was 6.812. The splicing mutation c.2427-2 A > G CADD score was 24, GERP score was 6.16, DANN score was 0.998, and the PhyloP100way vertebrate Score was 5.693. Q-PCR of the *PIGN* transcript revealed that levels of the patient mRNA were decreased compared to those in the normal control subjects (Figure 1D).

**TABLE 1:** Summary of all phosphatidylinositol-glycan biosynthesis class N (*PIGN*) mutations leading to multiple congenital anomalies-hypotonia-seizures syndrome 1 (MCAHS1).

	Mutation	Age	Sex	Birth		Dysmorphic features			Congenital anomalies			Neurologic						Brain MRI		
				weight	OFC	Palate	Ears	Fingers	Cardiac	Urinary	intestinal malrotation, anal stenosis or atresia	development delay	hypotonia	Nystagmus	Tremor	Seizure	Feeding	Corpus callosum	Cerebellar atrophy	Cerebral volume loss
Maydan et al., 2011	c.2126 G > A (p.R709Q)	29 months	M	3566	37	+	+	+	+	+	+	+	+	+	+	+	NR	−	−	+
		14 months	M	4065	37	+	+	+	+	+	+	+	+	+	+	−	NR			
		1 month	M	3850	35.5	NR	+	+	+	+	+	+	+	−	+	+	NR			
		5 months	F	3410	34.5	−	+	−	+	−	+	+	+	+	+	+	−	+	+	−
		3 months	F	4250	NR	−	+	−	−	−	−	+	+	+	+	+	−	+	−	+
		17 months	F	4300	NR	−	+	+	+	−	−	+	+	+	−	+	+			
		39 months	M	4800	NR	other	+	+	−	−	−	+	+	+	−	+	+			
Ohba et al., 2014	c.808 T > C; c.963 G > A	9 years	F	3390	35	+	+	+	−	+	+	+	+	+	+	+	+			
		2 years	M	3252	35	+	+	+	−	−	−	+	+	+	+	+	NR			
Brady et al., 2014	c.1574?1G > A	16 weeks	M	NA	NA	+	+	+	+	+	+	NA	NA	NA	NA	NA	NA			
Couser et al., 2015	c.406T > G; c.2576C > G	2 years	M	4271	36.8	NR	+	+	+	+	+	+	+	−	−	+	+			
Fleming et al., 2016	c.2340 T > A; c.1434 + 5 G > A	30 months	F	3350	35	+	+	−	−	−	+	+	+	+	−	+	+			
		18 months	F	3147	36.5	+	+	−	−	−	+	+	+	+	−	+	+			
	c.709 G > A; c.2411_2412delT AinsAG	14 years	F	2756	NA	+	−	−	−	−	−	+	+	−	−	+	−	+	−	+
	c.548_549?6del	4 months	F	4008	36	+	+	+	−	−	+	+	+	+	−	+	+		−	−
Khayat et al., 2016	c.755A > T	5 years	F	3300	NA	−	+	+	−	−	−	+	+	+	−	+	−	−	−	+
Nakagawa et al., 2016	c.808T > C	6 years	M	2880	33	−	−	−	−	−	−	+	+	+	−	−	−	−	+	−
Jezela-Stanek et al., 2016	c.790G > A; c.932T > G	2 months	F	4300	34	−	−	−	−	−	−	+	+	+	+	+	−	−	+	−
Pagnamenta et al., 2017	c.932T > G; c.694 > T	NA	F	NA	NA	NA	NA	−	NA	NA	NA	+	−	−	−	+	NA	−	−	+
Jiao et al., 2020	c.2122C > T; c.2557A > C	2 years	M	NA	NA	NA	NA	NA	−	−	−	+	+	−	NA	+	−	−	−	−
	c.2759_2760del; c.1172 + 1G > A	4 years 9 months	M	NA	NA	+	−	−	−	−	−	+	+	−	NA	+	−	−	−	−
	c.1109A > C; c.694A > T	3 years 6 months	M	NA	NA	−	−	−	−	−	−	+	+	−	NA	+	−	−	−	−
	c.1694G > A; c.2663 T > C;c.963G > A	2 years 8 months	F	NA	NA	+	−	+	−	−	−	+	+	−	NA	+	−	+	−	−
	c.343G > C; c.1694G > T	1 year 7 months	M	NA	NA	−	−	−	−	−	−	+	+	+	NA	+	−	−	−	−
	c.505C > T; c.769 T > G	2 years	M	NA	NA	−	−	−	−	−	−	+	−	−	NA	+	−	−	−	−
	c.895 C > T; c.629 T > C	2 years 5 months	F	NA	NA	−	−	−	−	−	−	+	+	−	NA	+	−	−	+	−

## Discussion

The MCAHS1 phenotype always includes congenital anomalies, seizures, developmental delay, and hypotonia; cerebellar atrophy, nystagmus, and diaphragmatic hernia may also be present (Table 2). The etiology of MCAHS1 involves *PIGN* mutations. Early onset and focal seizures appear to be a common feature of patients with *PIGN* mutation (Brady et al., 2014; Jiao et al., 2020). The clinical severity of cases is predicted to be associated with the severity of functional loss resulting from mutations in *PIGN*. Patients with biallelic truncated *PIGN* variants show a severe clinical phenotype, including early onset of intractable seizures and death *in utero* or shortly after birth, indicating that truncated variants are likely related to the severe phenotype (Thiffault et al., 2017). It is difficult to predict MCAHS1 by ultrasound examination because rare cases do not show abnormalities during gestation. However, in this study, the patient initially showed head and neck hygroma, which then disappeared. Additionally, the fetal system ultrasound displayed tetralogy of Fallot for which the phenotype spectrum was enriched during gestation. This finding enabled the prenatal diagnosis of MCAHS1. Eight genes (*PIGA*, *PIGL*, *PIGM*, *PIGN*, *PIGO*, *PIGV*, *PIGT*, and *PGAP2*) in this pathway are associated with neural abnormalities (Krawitz et al., 2012; Freeze, 2013; Hansen et al., 2013). Alkaline phosphatase (ALP) is a useful marker for suspected GPI anchor-synthesis pathway deficiencies caused by mutations in *PIGV*, *PIGO*, and *PGAP2*. Additionally, a recent study has reported that elevated ALP in patient serum may be useful in screening for *PIGN* mutations and MCAHS1 (Jiao et al., 2020).

**TABLE 2:** The allele frequencies in different databases of the two variants.

*PIGN* Variations	Allele frequency in gnomAD	Allele frequency in 1000 genomes	Allele frequency in ExAC	Allele frequency in dbSNP	Allele frequency in in-house database
NM_012327.6: c.2427-2A > G	0.00008806 (East Asian)	No records	No records	0.00003 (Asian)	No records
NM_012327.6: c.963G > A	0.001109 (East Asian)	No records	0.0012 (East Asian)	0.00 (East Asian)	0.00098232

Located on chromosome 18q21.33, *PIGN* is composed of 30 exons and encodes 931 amino acids (Hong et al., 1999). *PIGN* biallelic variants cause MCAHS1, which shows great clinical heterogeneity and autosomal recessive inheritance. Since PIGN is abundantly expressed in various tissues, mutation results in diverse phenotypes and involves various systems (Paulick and Bertozzi, 2008; Nakagawa et al., 2016). Additionally, *PIGN* mutations in a mouse model result in a holoprosencephaly like phenotype (McKean and Niswander, 2012). Yeast Mcd4 (a *PIGN* ortholog) mutants display defective bud emergence, polarized growth, marked morphological defects, and defective transportation from the endoplasmic reticulum to the Golgi specific for GPI-anchored proteins (Gaynor et al., 1999). The GPI backbone consists of alternating phosphoethanolamine and sugar moieties bound to phosphatidylinositol. *PIGN* is involved in GPI anchor biosynthesis; *PIGN* encodes GPI ethanolamine phosphate transferase, is expressed in the endoplasmic reticulum, and transfers phosphoethanolamine to the first mannose of the GPI anchor (Freeze et al., 2012). GPI anchors sort transport signals from the GPI-anchored protein at their site of synthesis, the endoplasmic reticulum, to their final destination, the cell surface, and enable GPI-anchored proteins to anchor in the cell membrane by covalent linkage to GPI (Kinoshita et al., 2008; Muñiz and Riezman, 2016). *PIGN* mutations lead to low expression of GPI-anchored proteins, which have roles in cell adhesion, signal transduction, and antigen presentation (Fujita and Jigami, 2008; Paulick and Bertozzi, 2008). Maydan et al. (2011) have reported that *PIGN* mutations lead to low expression of GPI-anchored proteins, particularly CD59, on the surface of patient fibroblasts.

In this study, the synonymous mutation c.963G > A was found in exon 10 of *PIGN*, which has been reported previously (Ohba et al., 2014). The synonymous mutation c.963G > A leads to abnormal transcription because this site is located at the last nucleotide of exon 10. The abnormal transcription contains two new sequences: one with a 53 bp insertion of intron 10 sequences and one with a 41 bp deletion of the entire exon 10, both leading to a frameshift mutation, and to two premature stop codons (p. Ala322Valfs^∗^24 and p. Glu308Glyfs^∗^2) (Ohba et al., 2014). The synonymous mutation c.963G > A results in extremely low expression of GPI-anchored proteins on patient granulocytes, specifically CD16 and CD24, suggesting severe and complete loss of PIGN activity. The degree of CD16 and CD24 deficiency is sufficient to cause severe neurological phenotypes (Ohba et al., 2014). c.2427-2 A > G is a splicing mutation in intron 25. This mutation may lead to intron loss, intron shortening, intron transfer to exons, effects on protein expression, and the production of a non-functional protein. The RT-PCR and Sanger sequencing results revealed that the mutation led to the skipping of exon 26 during transcription, and to the low expression of *PIGN*; possibly because the abnormal mRNA was degraded by non-sense-mediated mRNA decay.

In this study, two mutations in *PIGN* were identified. These findings provided reproduction guidance for this family, a basis for prenatal diagnosis, and broadened the gene and phenotype spectrum of MCAHS1.

## Data Availability Statement

The datasets for this article are not publicly available due to concerns regarding participant/patient anonymity. Requests to access the datasets should be directed to the corresponding author.

## Ethics Statement

The studies involving human participants were reviewed and approved by the Ethics Committee of Shengjing Hospital of China Medical University (ethics approval number: 2013PS33K). Written informed consent to participate in this study was provided by the participants’ legal guardian/next of kin. Written informed consent was obtained from the minor(s)’ legal guardian/next of kin for the publication of any potentially identifiable images or data included in this article.

## Author Contributions

SX and QN conceived and designed the experiments. ML, YL, and CL helped recruit the patients and their family members. CL, HH, and YL performed the experiments and helped with the genetic analysis. ML, YM, QN, and YL wrote the manuscript. All authors contributed to the article and approved the submitted version.

## Conflict of Interest

The authors declare that the research was conducted in the absence of any commercial or financial relationships that could be construed as a potential conflict of interest.

## Footnotes

1 http://www.hgmd.cf.ac.uk/ac/index.php

2 https://www.ncbi.nlm.nih.gov/clinvar/

3 http://bejerano.stanford.edu/MCAP/

4 https://sift.bii.a-star.edu.sg/

5 http://genetics.bwh.harvard.edu/pph2/

6 http://www.genetics.wustl.edu/jflab/lrt_query.html

7 http://www.mutationtaster.org/

8 https://cadd.gs.washington.edu/

9 http://fathmm.biocompute.org.uk/inherited.html

10 http://provean.jcvi.org/index.php
